# The True Antidote for Opium

**Published:** 1883-07

**Authors:** I. H. Stearns

**Affiliations:** Greenwood, Wis.


### ARTICLE VIII.

THE TRUE ANTIDOTE FOR OPIUM. By I. H. STEARNS, M.D

The great antidote for pain is opium in its various forms of preparation, and, conversely, the antidote for opium in overdoses is pain.

In practice, the dose of the opiate is graduated by the amount of the pain, which, being great and persistent or continuous, will carry off a large amount of the drug without affecting the nervous centers. So in cases where there has been exhibited a large and dangerous dose of the opiate, the indications are plainly to cause pain to the patient which shall be continuous and unrelenting.

The question as to a simple and exquisite torture that is un ceasing in its agonizing character, was long ago settled by the Inquisition, which resorted to the "thumb-screw." Any means by which a steady pressure upon the terminal branches of the nerves is made will answer the purpose. A hand vice upon each of the thumbs and a snap clothespin on each finger is most admirable and effective, and they should be kept on until they begin to feel painful, and then released one at a time.

Any physician can see the philosophy of the idea, and attention is called to it because only last night the writer had a case where the patient had purposly taken ten doses of morphine. In the absence of other facilities, twine was wound tightly around the terminal points of the thumbs and fingers, rendering them "black and blue," but they gave her no annoyance until after about ten hours, long previous to which time she was beyond danger. Le the physician remember that the real antidote to opium is pain and, as far as known, there is no other, notwithstanding th various drugs that have been from time to time fashionable in this regard, and many patients will be saved that would otherwise be lost.

Greenwood, Wis., May 20, 1883.

